# Respiratory adverse-event signals of four Bruton tyrosine kinase inhibitors: cross-database replication and temporal characterization in FAERS and JADER

**DOI:** 10.3389/fonc.2026.1998864

**Published:** 2026-10-22

**Authors:** Zihui Zheng, Fei Zhao, Zinan Zhao, Pengfei Jin

**Affiliations:** Department of Pharmacy, Beijing Hospital, National Center for Gerontology; National Clinical Research Center for Gerontology; The Key Laboratory of Geriatrics of the National Health Commission (NHC); Institute of Geriatric Medicine, Chinese Academy of Medical Sciences, Beijing, China

**Keywords:** Bruton tyrosine kinase inhibitors, disproportionality, FAERS, JADER, pharmacovigilance, pneumonia, respiratory adverse events, Weibull analysis

## Abstract

**Background:**

Broad pharmacovigilance profiles of Bruton tyrosine kinase (BTK) inhibitors have been described, but respiratory adverse events have not been systematically characterized across individual agents using independent reporting systems. We therefore focused on respiratory reporting phenotypes and their cross-database reproducibility.

**Methods:**

We analyzed spontaneous reports involving ibrutinib, acalabrutinib, zanubrutinib, and pirtobrutinib in FAERS and JADER. Preferred terms (PTs) within the MedDRA Respiratory, thoracic and mediastinal disorders system organ class were screened using the reporting odds ratio (ROR), which was prespecified as the primary disproportionality measure. A signal required at least three reports and a lower 95% confidence limit of the ROR >1. Cross-database replication required the same drug–PT combination to meet this criterion independently in both databases. Pneumonia was evaluated separately because it is formally classified under *Infections and infestations*. For pneumonia, PRR, BCPNN, and MGPS were added as supportive sensitivity analyses to assess signal robustness without altering the primary ROR classification. Time to onset was characterized using exact treatment-start and event-onset dates and Weibull models when sufficient data were available.

**Results:**

FAERS contained 84,217 target-drug reports and 255,010 adverse-event records, whereas JADER contained 1,368 reports and 2,035 adverse-event records. Within the respiratory SOC, 70 drug–PT combinations met the ROR criterion in FAERS and five in JADER. Three signals were replicated across databases, all involving ibrutinib: epistaxis, haemothorax, and pleural effusion. Pneumonia met the primary ROR criterion for all four agents in FAERS and for ibrutinib, acalabrutinib, and pirtobrutinib in JADER. Multi-algorithm agreement supported the robustness of the pneumonia signal for ibrutinib and pirtobrutinib in FAERS and for ibrutinib, acalabrutinib, and pirtobrutinib in JADER. In FAERS, pneumonia showed early-failure Weibull patterns for ibrutinib (β=0.55, 95% CI 0.53–0.58) and acalabrutinib (β=0.69, 95% CI 0.59–0.83); corresponding JADER estimates were compatible with random-failure patterns.

**Conclusions:**

Respiratory reporting patterns varied across BTK inhibitors and were only partly reproducible across FAERS and JADER. Cross-database replication was concentrated in selected ibrutinib-associated respiratory events, whereas pneumonia showed reproducible ROR signals for three agents. These findings characterize reporting patterns rather than incidence or comparative clinical risk.

## 1 Introduction

Bruton tyrosine kinase (BTK), a Tec-family non-receptor tyrosine kinase, is a central component of B-cell receptor signaling and contributes to B-cell proliferation, survival, migration, and adhesion. BTK inhibition has changed the treatment of chronic lymphocytic leukemia/small lymphocytic lymphoma (CLL/SLL), mantle cell lymphoma, Waldenström macroglobulinemia, and other B-cell malignancies. The clinical sequence from ibrutinib to the more selective covalent inhibitors acalabrutinib and zanubrutinib, and subsequently to the non-covalent inhibitor pirtobrutinib, has expanded treatment options after intolerance or resistance. These agents differ in binding mode, kinase selectivity, indications, treatment history, and cumulative post-marketing exposure, factors that may contribute to differences in reported adverse-event profiles (1, 2).

Respiratory complications are clinically relevant in patients receiving BTK inhibitors. The underlying hematologic malignancy, hypogammaglobulinemia, previous immunochemotherapy, corticosteroid exposure, cytopenias, and other host factors already increase susceptibility to pulmonary infection. In a 2025 meta-analysis of 18 clinical trials, pooled incidences of pneumonia during BTK inhibitor monotherapy for CLL were 13% for any-grade and 8% for grade 3 or higher events; no significant difference was detected among ibrutinib, acalabrutinib, and zanubrutinib, whereas pneumonia was more frequent in relapsed/refractory than treatment-naïve disease (3). A separate meta-analysis of randomized trials found no overall increase in infection with BTK inhibitor-containing regimens but identified increased pneumonia risk in some combination-treatment settings (4).

The pulmonary safety spectrum is not limited to conventional pneumonia. A 2026 meta-analysis of 88 studies involving 23,737 patients estimated a pooled prevalence of proven or probable invasive fungal infection of 2.83%, with aspergillosis the most frequently reported fungal infection and corticosteroid exposure and multiple previous treatment lines among the main risk factors (5). Experimental work also supports a role for BTK-dependent myeloid-cell signaling in antifungal host defense (6). In parallel, BTK and related Tec-family kinases participate in platelet activation downstream of collagen receptors such as glycoprotein VI. Their inhibition can impair platelet function, providing biological context for bleeding manifestations that may present within the respiratory system, including epistaxis, haemoptysis, and haemothorax (7, 8).

Recent post-marketing studies have characterized broad BTK inhibitor safety patterns. A 2025 FAERS/VigiBase analysis of ibrutinib, acalabrutinib, and zanubrutinib identified substantial inter-drug heterogeneity across SOC, standardized MedDRA query, and PT analyses, including infection- and haemorrhage-related domains (9). A newly published 2026 FAERS study extended broad safety profiling to all four agents and described generation-specific reporting patterns and temporal distributions, including pneumonia (10). These studies were not designed as a respiratory-focused dual-database evaluation that separates formal respiratory-SOC events from clinically important pulmonary events outside that SOC.

Unlike recent broad-spectrum pharmacovigilance studies of BTK inhibitors, our analysis was designed around a respiratory-specific question. We used ROR as a common primary metric to permit PT-level screening and predefined cross-database replication between FAERS and JADER, while reserving multi-algorithm assessment for the prespecified clinically important pneumonia endpoint.

## 2 Materials and methods

### 2.1 Study design and data sources

This retrospective pharmacovigilance study used spontaneous adverse-event reports from the U.S. Food and Drug Administration Adverse Event Reporting System (FAERS) and the Japanese Adverse Drug Event Report (JADER) database. Four BTK inhibitors were evaluated: ibrutinib, acalabrutinib, zanubrutinib, and pirtobrutinib. FAERS and JADER were processed and analyzed independently; results were compared descriptively rather than pooled. The FAERS source data extended through 2026 Q2, while the JADER processing logs applied a 2004–2026 calendar-year filter. The design followed a two-stage structure: construction of database-wide background analytic datasets followed by identification of target-drug analytic cohorts.

### 2.2 Data processing and construction of background analytic databases

For FAERS, the DEMO, DRUG, REAC, OUTC, INDI, THER, and PRSR tables were linked using report identifiers. Duplicate case reports were removed at the case level by retaining the most recent version according to FDA_DT and, when dates were tied, the record with the largest PRIMARYID. According to the processing logs, the source DEMO table contained 24,680,712 records before deduplication. The cleaned FAERS background analytic database contained 20,586,679 DEMO records, 74,367,111 DRUG records, and 61,152,348 REAC records. For JADER, demographic, drug, adverse-event, and related tables were integrated using unique report identifiers; the processed background database contained 1,028,464 reports, 4,936,577 drug records, and 1,676,290 adverse-event records. These background datasets served as reference populations for case–non-case disproportionality analyses.

### 2.3 Identification of target-drug reports

Target drugs were identified using standardized generic names and prespecified drug-name variants. In FAERS, analyses were restricted to reports in which the target BTK inhibitor was designated as the primary suspect drug (role_cod = PS). This yielded 70,195 reports for ibrutinib, 10,598 for acalabrutinib, 2,861 for zanubrutinib, and 563 for pirtobrutinib, corresponding to 219,174, 25,890, 8,772, and 1,174 adverse-event records, respectively. In JADER, analyses were restricted to records in which the target BTK inhibitor was classified as a suspected drug. This yielded 1,065, 166, 54, and 83 reports, corresponding to 1,631, 231, 58, and 115 adverse-event records, respectively (**Figure 1**).

**Figure 1:**
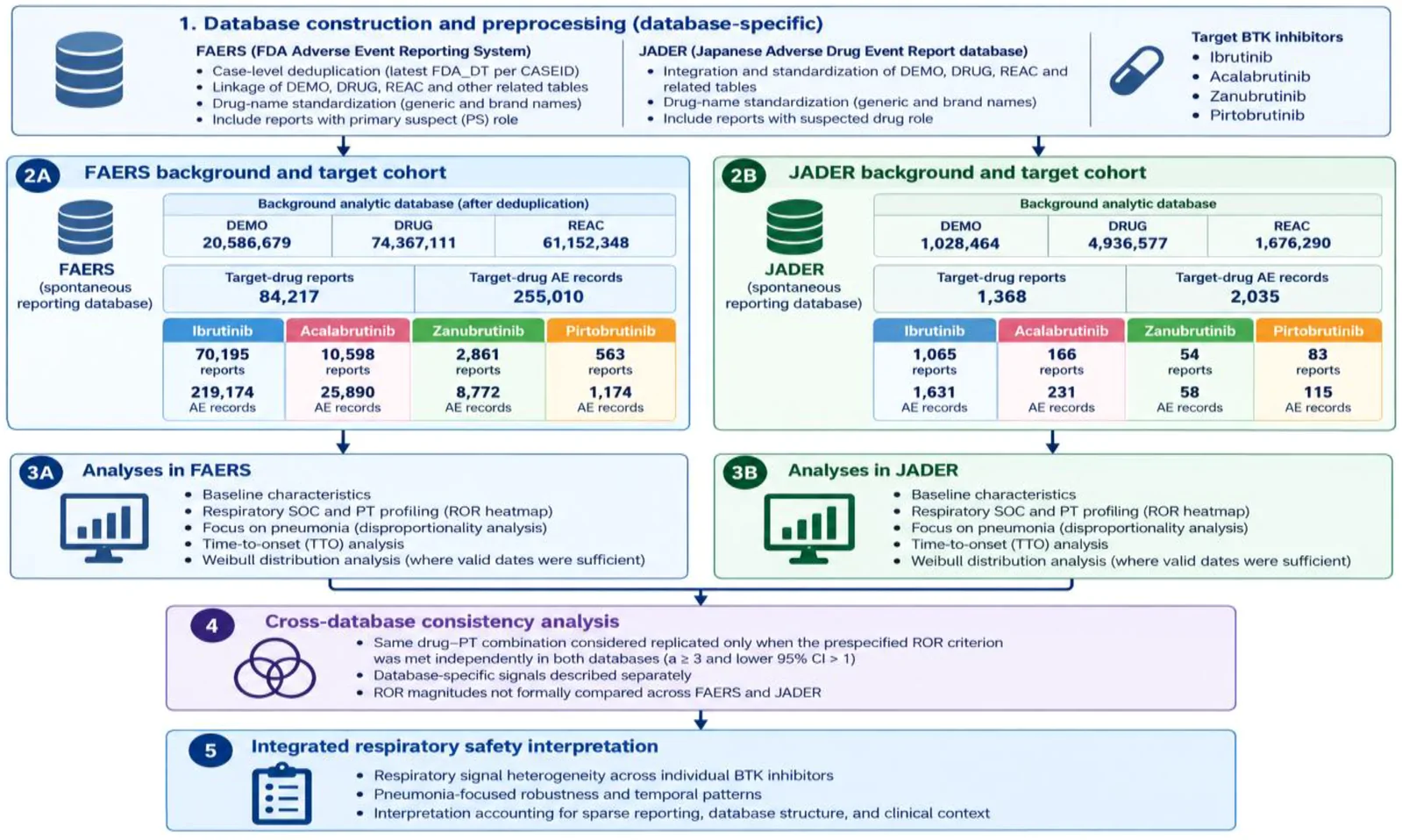
Study workflow for the comparative analysis of respiratory adverse events associated with four Bruton tyrosine kinase inhibitors in FAERS and JADER. The background analytic databases were constructed before target-drug selection; FAERS target reports were restricted to primary-suspect drugs and JADER target reports to suspected drugs. Analyses were performed independently before cross-database interpretation.

### 2.4 Definition of respiratory adverse events

Adverse events were standardized according to the Medical Dictionary for Regulatory Activities (MedDRA) at the preferred term (PT) and system organ class (SOC) levels. The primary respiratory safety-profile analysis included PTs classified under the MedDRA SOC Respiratory, thoracic and mediastinal disorders. PT-level analyses were conducted separately for each BTK inhibitor in FAERS and JADER. Because clinically relevant respiratory complications are not necessarily assigned to the respiratory SOC, prespecified pulmonary events were additionally assessed using their exact MedDRA PTs. Pneumonia was evaluated as a clinically important respiratory infection although the PT Pneumonia is formally classified under Infections and infestations. This distinction was retained throughout the analysis. Respiratory bleeding-related PTs, including Epistaxis, Haemoptysis, and Haemothorax, were included when formally mapped to the respiratory SOC.

### 2.5 Primary ROR-based disproportionality analysis

The reporting odds ratio (ROR) was prespecified as the primary disproportionality measure for the respiratory safety-profile analysis. RORs and corresponding 95% confidence intervals (CIs) were calculated separately for each drug–PT combination in FAERS and JADER. A positive ROR signal was defined as at least three reports (a ≥3) and a lower 95% CI greater than 1. This primary ROR framework was used consistently for respiratory PT screening, heatmap visualization, signal counting, and cross-database replication. Because this analysis involved exploratory screening across numerous PTs, no formal adjustment for multiple testing was applied. Accordingly, the identified signals should be interpreted as report-level disproportionality findings warranting further evaluation rather than as definitive evidence of clinical risk.

### 2.6 Respiratory PT profile and heatmap analysis

All PTs mapped to Respiratory, thoracic and mediastinal disorders were first identified for each drug and database. To permit comparison without overcrowding, the heatmap displayed the 30 PTs with the highest combined reporting frequencies across the four BTK inhibitors and both databases. Each cell displayed the ROR and 95% CI; an asterisk denoted fulfillment of the predefined ROR criterion. Unreported or non-evaluable drug–PT combinations were displayed as unavailable. For visualization, the color scale was capped at ROR = 20 on a logarithmic scale, while numerical annotations retained the actual estimates.

### 2.7 Focused pneumonia analysis and multi-algorithm robustness assessment

Pneumonia was prespecified as a clinically important respiratory event and was evaluated separately using the exact MedDRA PT Pneumonia, which is formally classified under Infections and infestations rather than Respiratory, thoracic and mediastinal disorders.

The ROR served as the primary signal measure for pneumonia, consistent with the main respiratory analysis. To assess the robustness of the pneumonia signal across alternative disproportionality approaches, three additional algorithms—proportional reporting ratio (PRR), Bayesian confidence propagation neural network (BCPNN), and multi-item gamma Poisson shrinker (MGPS)—were applied as supportive sensitivity analyses. These additional algorithms were not used to redefine the primary respiratory signal set or the cross-database replication criterion.

PRR positivity required a≥3, PRR≥2, and χ^2^ ≥4; BCPNN positivity required IC025 >0; and MGPS positivity required EBGM05>2. Agreement across the four methods was interpreted as evidence of greater signal robustness, whereas discordance among algorithms was retained and reported rather than used to override the primary ROR classification (11–13).

### 2.8 Cross-database consistency analysis

Cross-database replication was assessed exclusively using the prespecified primary ROR criterion. A drug–PT combination was classified as replicated only when a ≥3 and the lower 95% CI of the ROR was >1 independently in both FAERS and JADER. Results from PRR, BCPNN, and MGPS were not used to determine cross-database replication.

### 2.9 Descriptive analysis

Baseline characteristics of target-drug reports were summarized separately for each BTK inhibitor within FAERS and JADER. Variables included the number of target-drug reports, number of target-drug adverse-event records, reporting period, reporting year, sex, age, and reporter type. Categorical variables were summarized as frequencies and percentages. For FAERS, age was categorized *a priori* as <60 years, 60–74 years, 75–89 years, ≥90 years, or missing. Because JADER commonly reports age by decade, decade-based categories were harmonized to the same broad age groups where assignment was reliable; non-specific descriptors such as adult or elderly were classified as missing. Reporter type was classified as physician, pharmacist, other healthcare professional, consumer, or other. JADER reports containing multiple reporter qualifications were assigned to the other category to maintain mutually exclusive categories and prevent double counting.

### 2.10 Time-to-onset analysis

For the focused temporal analysis, time to onset (TTO) was evaluated among pneumonia reports with complete and valid treatment-start and event-onset dates. In FAERS, the target-drug treatment start date was obtained from THER and linked to the report-level event date using report and drug-sequence identifiers. In JADER, the target-drug administration start date and adverse-event onset date were obtained from DRUG and REAC. TTO was defined as the interval in days between treatment initiation and event onset; same-day events were assigned a TTO of 1 day. Reports were excluded when either date was missing, only year or year-month information was available, dates were internally inconsistent, or the calculated interval was negative. Incomplete dates were not imputed. TTO was summarized using median, interquartile range (IQR), and minimum-to-maximum range and was interpreted only among analyzable reports.

### 2.11 Weibull distribution analysis

Weibull distribution analysis was used to characterize pneumonia onset patterns when sufficient valid TTO observations were available. The distribution was fitted by maximum likelihood estimation, and scale (alpha) and shape (beta) parameters were estimated for each drug–database combination. Bootstrap resampling (2,000 iterations) was used to derive 95% CIs. An early-failure pattern was defined when the upper 95% CI of beta was <1, a random-failure pattern when the 95% CI included 1, and a wear-out pattern when the lower 95% CI was >1. Estimates based on very small samples were treated as exploratory or were not formally classified. These patterns describe reported onset among analyzable cases and do not estimate a population hazard function.

## 3 Results

### 3.1 Data extraction and baseline characteristics

After data cleaning, standardization, and role restriction, 84,217 target-drug reports were identified in FAERS: 70,195 for ibrutinib, 10,598 for acalabrutinib, 2,861 for zanubrutinib, and 563 for pirtobrutinib. These reports corresponded to 255,010 target-drug adverse-event records. JADER contained 1,368 target-drug reports (1,065, 166, 54, and 83, respectively) and 2,035 target-drug adverse-event records.

The two databases differed in demographic completeness and reporting source. In JADER, patients aged 75–89 years comprised more than half of reports for all four agents (51.9%–62.7%). Age was missing in 52.2%–70.5% of FAERS reports across the four drugs. FAERS also had a substantial consumer-reporting component, whereas JADER reports for ibrutinib, acalabrutinib, and zanubrutinib were predominantly submitted by physicians. Baseline characteristics are summarized in **Table 1**.

**Table 1:** Baseline characteristics of target-drug reports in FAERS and JADER.

Characteristic	FAERS				JADER			
Characteristic	Ibrutinib	Acalabrutinib	Zanubrutinib	Pirtobrutinib	Ibrutinib	Acalabrutinib	Zanubrutinib	Pirtobrutinib
Overall								
Target-drug reports, n	70,195	10,598	2,861	563	1,065	166	54	83
Target-drug adverse-event records, n	219,174	25,890	8,772	1,174	1,631	231	58	115
Reporting period; peak year, n (%)	2013–2026; peak 2022: 10,997 (15.7)	2017–2026; peak 2025: 2,575 (24.3)	2019–2026; peak 2025: 851 (29.7)	2023–2026; peak 2025: 203 (36.1)	2014–2025; peak 2023: 147 (13.8)	2021–2025; peak 2024: 58 (34.9)	2025; peak 2025: 54 (100.0)	2024–2025; peak 2025: 64 (77.1)
Demographic characteristics								
Male	40,070 (57.1)	5,512 (52.0)	299 (10.5)	287 (51.0)	677 (63.6)	87 (52.4)	39 (72.2)	53 (63.9)
Female	25,549 (36.4)	3,353 (31.6)	225 (7.9)	156 (27.7)	289 (27.1)	62 (37.3)	12 (22.2)	12 (14.5)
Missing sex	4,576 (6.5)	1,733 (16.4)	2,337 (81.7)	120 (21.3)	99 (9.3)	17 (10.2)	3 (5.6)	18 (21.7)
<60 years	4,089 (5.8)	288 (2.7)	114 (4.0)	30 (5.3)	151 (14.2)	14 (8.4)	0 (0.0)	4 (4.8)
60–74 years	14,290 (20.4)	1,411 (13.3)	339 (11.8)	111 (19.7)	192 (18.0)	32 (19.3)	7 (13.0)	9 (10.8)
75–89 years	13,240 (18.9)	1,672 (15.8)	365 (12.8)	115 (20.4)	592 (55.6)	90 (54.2)	28 (51.9)	52 (62.7)
≥90 years	1,152 (1.6)	181 (1.7)	25 (0.9)	13 (2.3)	13 (1.2)	2 (1.2)	1 (1.9)	5 (6.0)
Missing age	37,424 (53.3)	7,046 (66.5)	2,018 (70.5)	294 (52.2)	117 (11.0)	28 (16.9)	18 (33.3)	13 (15.7)
Report characteristics								
Physician	11,919 (17.0)	2,212 (20.9)	997 (34.8)	61 (10.8)	930 (87.3)	143 (86.1)	37 (68.5)	0 (0.0)
Pharmacist	1,618 (2.3)	286 (2.7)	300 (10.5)	23 (4.1)	74 (6.9)	10 (6.0)	10 (18.5)	0 (0.0)
Other healthcare professional	6,018 (8.6)	580 (5.5)	272 (9.5)	30 (5.3)	20 (1.9)	4 (2.4)	1 (1.9)	0 (0.0)
Consumer	46,642 (66.4)	4,271 (40.3)	665 (23.2)	335 (59.5)	15 (1.4)	2 (1.2)	0 (0.0)	0 (0.0)
Other	3,998 (5.7)	3,249 (30.7)	627 (21.9)	114 (20.2)	26 (2.4)	7 (4.2)	6 (11.1)	83 (100.0)

Percentages are based on target-drug reports. For JADER, decade-based age categories were harmonized using prespecified mappings; non-specific age descriptions were treated as missing. Reports with multiple JADER reporter qualifications were classified as other. AE, adverse event; FAERS, FDA Adverse Event Reporting System; JADER, Japanese Adverse Drug Event Report database.

### 3.2 Respiratory adverse-event signal profiles

The respiratory adverse-event profiles differed across the four BTK inhibitors and between FAERS and JADER (**Figure 2**). In FAERS, ibrutinib had the broadest respiratory-SOC signal profile. Among the displayed PTs, the largest RORs included Haemothorax (ROR 14.96, 95% CI 12.67–17.66), Pleural effusion (5.31, 5.00–5.63), Epistaxis (3.58, 3.36–3.82), Pulmonary mass (3.26, 2.80–3.80), Pulmonary oedema (3.14, 2.87–3.43), and Haemoptysis (2.96, 2.64–3.33). Pneumonitis also met the ROR criterion (2.35, 2.06–2.68).

**Figure 2:**
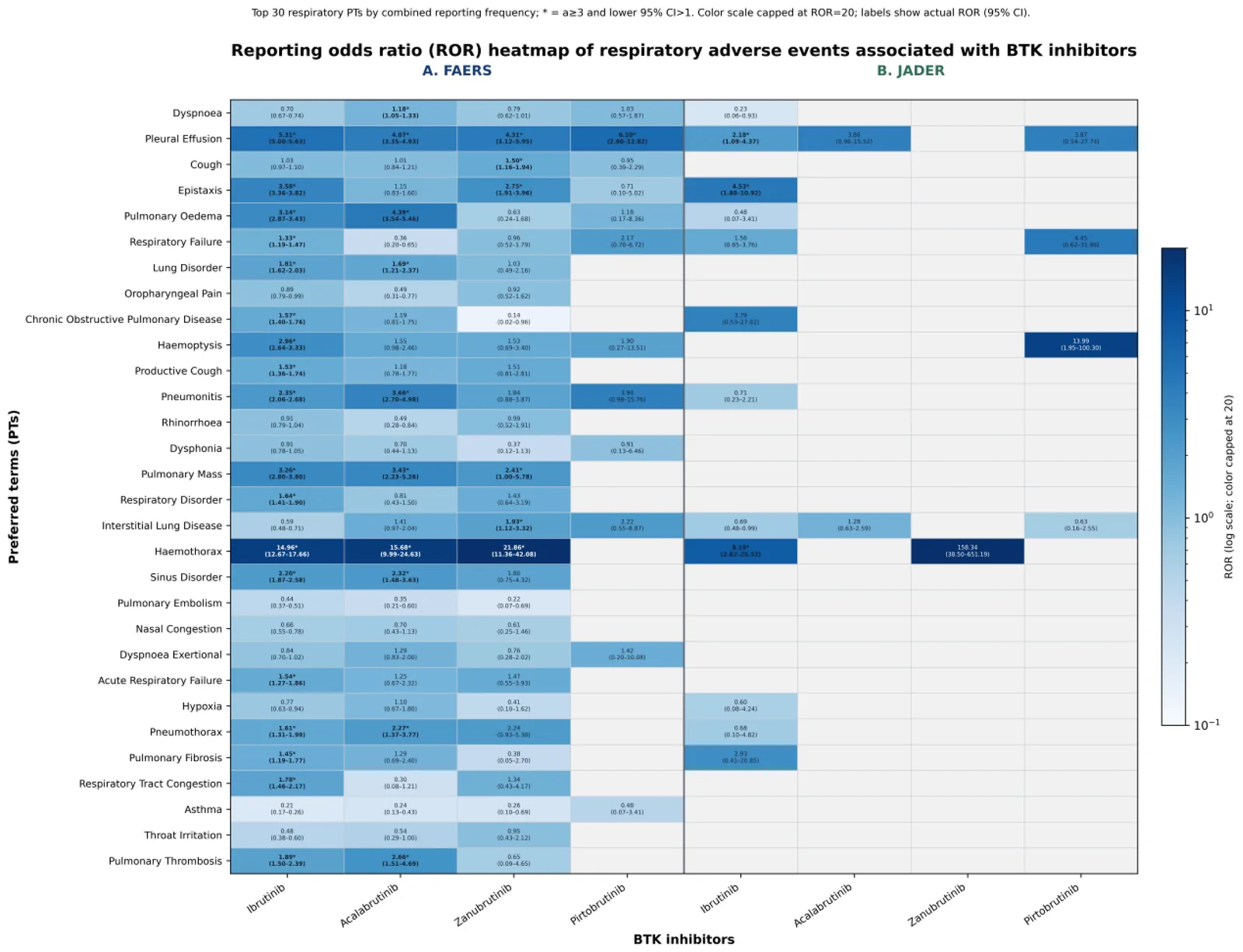
Reporting odds ratio heatmap of respiratory adverse events associated with BTK inhibitors in FAERS and JADER. The heatmap shows the 30 respiratory PTs with the highest combined reporting frequencies. Asterisks indicate a≥3 and lower 95% CI of the ROR >1. The color scale is logarithmic and capped at ROR = 20 for visualization; cell annotations retain the actual ROR and 95% CI. Grey cells indicate unavailable or non-evaluable combinations. ROR represents reporting disproportionality, not incidence or comparative risk.

Acalabrutinib showed positive signals for Haemothorax (15.68, 9.99–24.63), Pulmonary oedema (4.39, 3.54–5.46), Pleural effusion (4.07, 3.35–4.93), Pneumonitis (3.66, 2.70–4.98), and Pulmonary mass (3.43, 2.23–5.26), among others. Zanubrutinib showed a more restricted profile, with positive signals including Haemothorax (21.86, 11.36–42.08), Pleural effusion (4.31, 3.12–5.95), Epistaxis (2.75, 1.91–3.96), Interstitial lung disease (1.93, 1.12–3.32), and Cough (1.50, 1.16–1.94). For pirtobrutinib, Pleural effusion was the only displayed PT meeting the ROR criterion (6.10, 2.90–12.82).

JADER contained far fewer evaluable respiratory drug–PT pairs. For ibrutinib, Pleural effusion (2.18, 1.09–4.37), Epistaxis (4.53, 1.88–10.92), and Haemothorax (8.18, 2.62–25.52) met the ROR criterion in both databases. Several large JADER point estimates were based on sparse counts. Zanubrutinib-associated Haemothorax had an ROR of 158.34 (38.50–651.19) but was based on two reports; pirtobrutinib-associated Haemoptysis had an ROR of 13.99 (1.95–100.30) based on one report. Neither met the prespecified minimum-report criterion.

### 3.3 Pneumonia as a clinically important respiratory event

Pneumonia was reported in 2,760, 252, 86, and 29 adverse-event records for ibrutinib, acalabrutinib, zanubrutinib, and pirtobrutinib, respectively, in FAERS; corresponding JADER counts were 88, 16, 1, and 7. The proportions of pneumonia among target-drug adverse-event records were 1.26%, 0.97%, 0.98%, and 2.47% in FAERS and 5.40%, 6.93%, 1.72%, and 6.09% in JADER, respectively. These percentages describe reporting composition rather than incidence.

All four agents met the ROR criterion for pneumonia in FAERS. Ibrutinib and pirtobrutinib met all four algorithm-specific criteria, whereas acalabrutinib and zanubrutinib met the ROR and BCPNN criteria but not the prespecified PRR or MGPS criteria. In JADER, ibrutinib, acalabrutinib, and pirtobrutinib met all four criteria. Zanubrutinib met none; only one pneumonia record was available (**Table 2**).

**Table 2:** Disproportionality analysis of pneumonia associated with BTK inhibitors in FAERS and JADER.

Signal metric	FAERS				JADER			
Signal metric	Ibrutinib	Acalabrutinib	Zanubrutinib	Pirtobrutinib	Ibrutinib	Acalabrutinib	Zanubrutinib	Pirtobrutinib
Pneumonia records, n	2,760	252	86	29	88	16	1	7
ROR (95% CI)	2.46 (2.37–2.56)	1.89 (1.67–2.14)	1.90 (1.54–2.35)	4.86 (3.36–7.03)	4.52 (3.64–5.60)	5.88 (3.54–9.77)	1.38 (0.19–10.00)	5.12 (2.38–10.99)
PRR (chi-square)	2.44 (2344.91)	1.88 (104.18)	1.89 (36.39)	4.77 (86.81)	4.33 (226.99)	5.54 (60.24)	1.38 (0.11)	4.87 (21.77)
EBGM (EBGM05)	2.43 (2.34)	1.88 (1.66)	1.89 (1.53)	4.77 (3.30)	4.31 (3.48)	5.54 (3.33)	1.38 (0.19)	4.87 (2.27)
IC (IC025)	1.28 (1.23)	0.91 (0.72)	0.92 (0.59)	2.25 (1.55)	2.11 (1.74)	2.47 (1.40)	0.46 (−1.87)	2.28 (0.65)
**Positive algorithms, n/4**	**4/4**	**2/4**	**2/4**	**4/4**	**4/4**	**4/4**	**0/4**	**4/4**

Positive criteria: ROR, a≥3 and lower 95% CI>1; PRR, a≥3, PRR≥2 and chi-square≥4; BCPNN, IC025>0; MGPS, EBGM05>2. CI, confidence interval; EBGM, empirical Bayes geometric mean; IC, information component; PRR, proportional reporting ratio; ROR, reporting odds ratio. All disproportionality metrics in this table are derived from spontaneous reporting data and reflect the strength of reporting signals rather than actual clinical incidence or risk.

Bold values indicate that the corresponding algorithm-specific signal-detection criterion was met.

### 3.4 Cross-database consistency of respiratory signals

Within Respiratory, thoracic and mediastinal disorders, 70 drug–PT combinations met the ROR criterion in FAERS (47 for ibrutinib, 15 for acalabrutinib, seven for zanubrutinib, and one for pirtobrutinib), whereas five met the criterion in JADER, all involving ibrutinib. Three combinations were replicated across databases: ibrutinib–Epistaxis, ibrutinib–Haemothorax, and ibrutinib–Pleural effusion (**Figure 3**). Organising pneumonia and Pneumomediastinum associated with ibrutinib met the criterion only in JADER.

**Figure 3:**
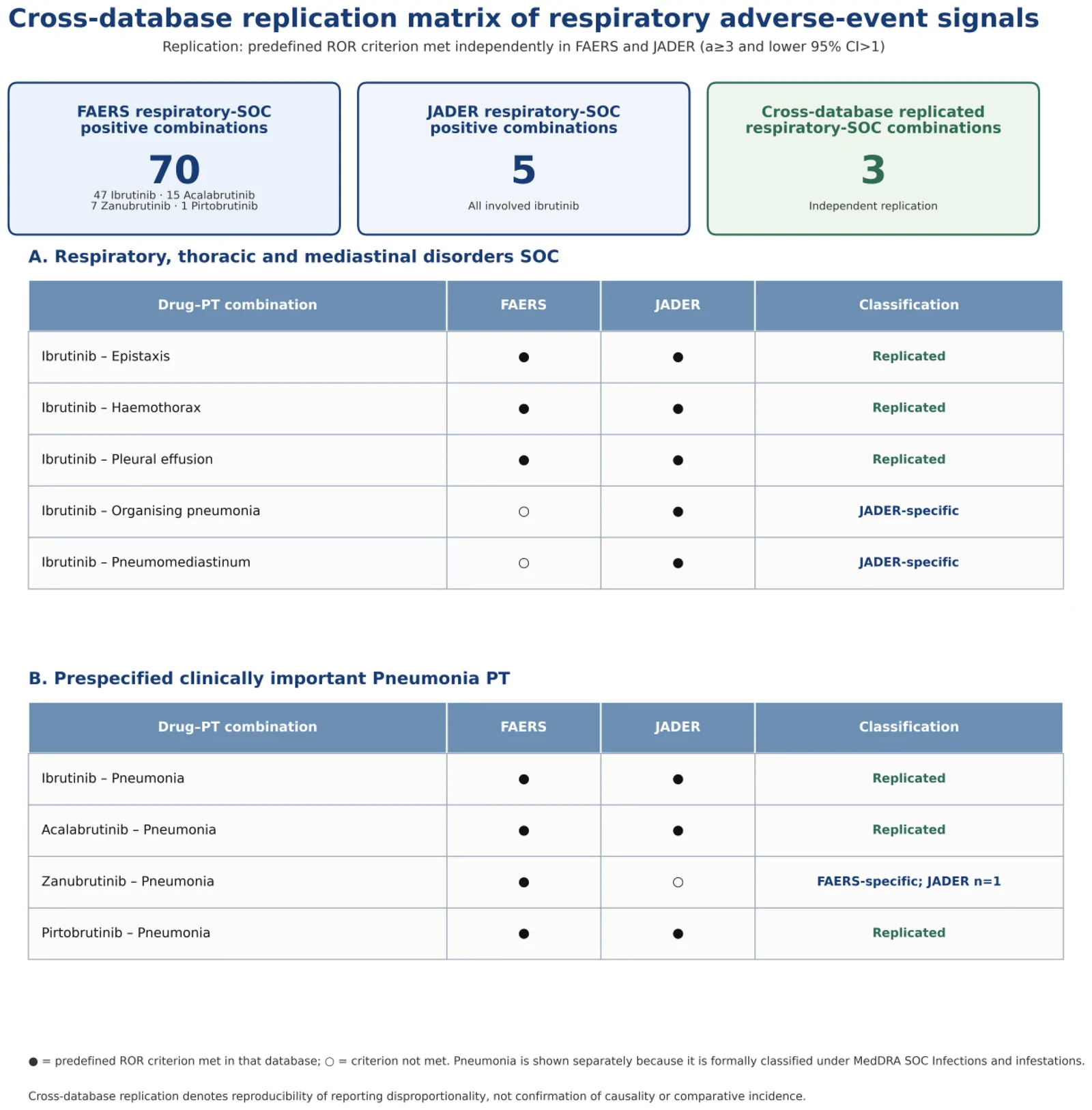
Cross-database replication matrix of respiratory adverse-event signals associated with BTK inhibitors in FAERS and JADER. A filled circle indicates that the predefined ROR criterion was met in that database; an open circle indicates that it was not. Pneumonia is shown separately because it is formally classified under the MedDRA SOC Infections and infestations. Cross-database replication denotes reproducibility of reporting disproportionality and does not establish causality or comparative incidence. **(A)** Respiratory, thoracic and mediastinal disorders system organ class (SOC) signals. **(B)** The prespecified clinically important Pneumonia preferred term (PT), which is formally classified under the MedDRA SOC Infections and infestations.

Pneumonia was evaluated separately because it is formally assigned to Infections and infestations. The pneumonia ROR signal was replicated for ibrutinib, acalabrutinib, and pirtobrutinib. Zanubrutinib met the criterion in FAERS but not JADER, where only one pneumonia report was available. Thus, lack of replication for newer agents was interpreted in the context of sparse JADER reporting rather than as evidence of no association.

### 3.5 Time-to-onset and Weibull analysis

Valid pneumonia TTO data were available for 678 FAERS and 36 JADER ibrutinib reports, 51 FAERS and seven JADER acalabrutinib reports, 13 FAERS and no JADER zanubrutinib reports, and two FAERS and four JADER pirtobrutinib reports. Formal Weibull interpretation was therefore concentrated on ibrutinib and acalabrutinib; the zanubrutinib FAERS estimate was considered exploratory, and pirtobrutinib data were insufficient for stable modelling.

For ibrutinib, the median TTO in FAERS was 114.5 days (IQR 20.0–475.8; range 1–3865). Weibull fitting yielded alpha=226.01 (95% CI 195.22–261.24) and beta=0.55 (95% CI 0.53–0.58), an early-failure pattern (**Figure 4A**). In JADER, median TTO was 57.5 days (IQR 32.8–208.8; range 2–1057), with alpha=132.97 (83.80–205.19) and beta=0.79 (0.67–1.05), a random-failure pattern (**Figure 4B**).

**Figure 4:**
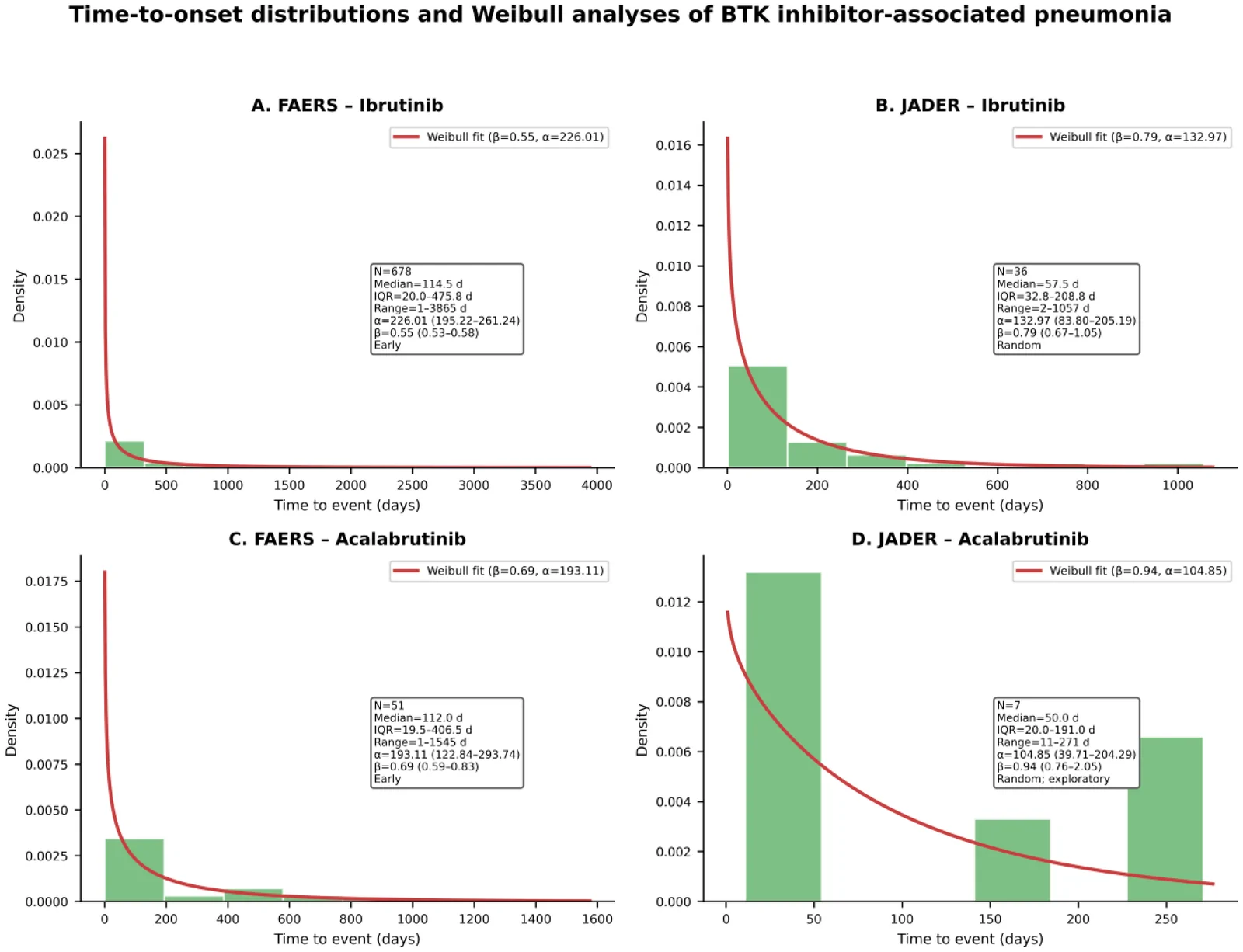
Time-to-onset distributions and Weibull analyses of pneumonia associated with ibrutinib and acalabrutinib in FAERS and JADER. **(A)** Ibrutinib in FAERS; **(B)** ibrutinib in JADER; **(C)** acalabrutinib in FAERS; **(D)** acalabrutinib in JADER. Solid red curves are fitted Weibull distributions. An early-failure pattern was defined when the upper 95% CI of beta was <1; random failure when the 95% CI included 1; and wear-out when the lower 95% CI was >1. The JADER acalabrutinib estimate is exploratory because only seven reports had valid dates.

For acalabrutinib, median TTO in FAERS was 112.0 days (IQR 19.5–406.5; range 1–1545), with alpha=193.11 (122.84–293.74) and beta=0.69 (0.59–0.83), also indicating early failure (**Figure 4C**). In JADER, seven reports were evaluable; median TTO was 50.0 days (IQR 20.0–191.0; range 11–271), alpha=104.85 (39.71–204.29), and beta=0.94 (0.76–2.05), corresponding to a random pattern. This JADER estimate was treated as exploratory (**Figure 4D**). Zanubrutinib in FAERS had 13 evaluable reports (median 38.0 days; beta=0.52, 95% CI 0.42–0.83); although statistically classified as early failure, the small sample warranted exploratory interpretation.

## 4 Discussion

This analysis identified a heterogeneous respiratory reporting profile across four BTK inhibitors and two spontaneous-reporting systems. Pneumonia was the most reproducible clinically important event: its ROR signal was present for all four agents in FAERS and for ibrutinib, acalabrutinib, and pirtobrutinib in JADER. Within the formal respiratory SOC, cross-database replication was more selective and was confined to ibrutinib-associated epistaxis, haemothorax, and pleural effusion. The temporal analysis added a separate dimension: pneumonia reports for ibrutinib and acalabrutinib in FAERS showed early-failure patterns, whereas corresponding JADER estimates were random and, for acalabrutinib, based on few evaluable reports. These findings support a respiratory safety profile that is mixed in phenotype and strongly influenced by drug, database, and report availability rather than a uniform class-wide pattern.

### 4.1 Pneumonia: a reproducible signal with heterogeneous causes

The pneumonia findings are consistent with clinical evidence that infection remains important during BTK inhibitor therapy, but spontaneous-reporting disproportionality should not be equated with incidence. The 2025 meta-analysis by Mahadevia et al. reported pooled incidences of 13% for any-grade and 8% for grade 3 or higher pneumonia during BTK inhibitor monotherapy in CLL, without a statistically significant difference among ibrutinib, acalabrutinib, and zanubrutinib (3). Pneumonia was more common in relapsed/refractory than treatment-naïve CLL, emphasizing the contribution of disease state and treatment history. Buchrits et al. likewise found no overall increase in infections with BTK inhibitor-containing regimens across randomized trials, although pneumonia was increased in some BTK inhibitor plus anti-CD20 comparisons (4).

The larger RORs observed for some drugs in our datasets, including pirtobrutinib, therefore cannot be used to rank absolute pneumonia risk. Pirtobrutinib has a shorter post-marketing history and substantially fewer reports than ibrutinib. In BRUIN CLL-321, pneumonia remained a clinically relevant treatment-emergent event, but those trial data do not establish a uniquely greater pneumonia risk for pirtobrutinib (14). Differences in treatment line, prior covalent BTK inhibitor exposure, background immune dysfunction, and reporting intensity are plausible contributors to the post-marketing pattern. The comparatively high FAERS pneumonia ROR for pirtobrutinib (4.86) should also be considered in the context of recent market entry and treatment channeling. Its initial US approval was for relapsed or refractory mantle cell lymphoma after at least two prior systemic therapies, including a BTK inhibitor (15). Such heavily pretreated patients may have substantial baseline infection susceptibility, while heightened attention to a newer agent may influence reporting intensity. Although early post-marketing reporting is sometimes described in terms of the Weber effect, an analysis of 62 drugs did not identify a consistent general Weber pattern in modern FAERS data (16). These factors remain possible contributors to the observed ROR; they cannot be disentangled or quantified in the present analysis.

There is biological plausibility for infection during BTK inhibition, but the mechanism is not specific to the MedDRA PT Pneumonia. BTK participates in normal B-cell and myeloid-cell function, while patients with B-cell malignancies may already have hypogammaglobulinemia, impaired cellular immunity, and treatment-related cytopenias. In mechanistic experiments, BTK inhibition impaired several neutrophil effector functions against Aspergillus fumigatus (6). A 2026 meta-analysis reported a 2.83% pooled prevalence of proven/probable invasive fungal infection and identified corticosteroid exposure and at least three previous treatment lines as risk factors (5). These data provide context for infection susceptibility but do not imply that the pneumonia reports in FAERS or JADER were fungal. The PT Pneumonia does not specify pathogen, radiographic pattern, microbiologic confirmation, or aspiration status, and should be interpreted as a broad reported clinical phenotype.

### 4.2 Respiratory bleeding signals and platelet biology

The PTs Epistaxis, Haemoptysis, and Haemothorax formed a distinct haemorrhagic component of the respiratory profile. Haemothorax showed high RORs for ibrutinib, acalabrutinib, and zanubrutinib in FAERS, while ibrutinib–Haemothorax and ibrutinib–Epistaxis were replicated in JADER. This pattern is pharmacologically plausible. BTK and TEC contribute to platelet signaling downstream of GPVI, and inhibition can reduce collagen-dependent platelet activation and aggregation (7). Ibrutinib has broader off-target kinase activity than later-generation agents, although platelet effects are not restricted to ibrutinib. A 2025 platelet-function study found marked inhibition of collagen-induced aggregation across BTK inhibitors, supporting a class component while also showing differences among agents (8).

This mechanism is supportive rather than causal evidence for the respiratory bleeding signals. Haemothorax in patients with hematologic malignancy can also follow thrombocytopenia, anticoagulant or antiplatelet treatment, pleural malignancy, infection, trauma, or invasive procedures. A recent FAERS/VigiBase study similarly identified haemorrhagic safety signals across BTK inhibitors and highlighted the relevance of concomitant antithrombotic therapy (9). The extreme JADER ROR for zanubrutinib-associated haemothorax is a useful example of the limits of point estimates: it was based on only two reports and failed the prespecified minimum-count criterion despite a lower confidence bound above 1.

### 4.3 Non-infectious pulmonary events were heterogeneous

Pleural effusion, pneumonitis, pulmonary oedema, pulmonary mass, and interstitial lung disease were not distributed uniformly across drugs or databases. These PTs represent different pathophysiologic processes and should not be collapsed into a single construct of ‘BTK inhibitor lung toxicity’. Pleural effusion may arise from infection, malignancy, heart failure, hemorrhage, or hypoalbuminemia; pulmonary oedema may be cardiovascular or fluid-related; pneumonitis and interstitial lung disease raise a different differential diagnosis that includes inflammatory drug injury and infection; and pulmonary mass may reflect underlying malignancy. Spontaneous reports rarely contain enough clinical detail to resolve these competing explanations. Their main value here is to identify reporting patterns that merit clinical review and, where reproducible, targeted follow-up.

### 4.4 Drug differences should not be interpreted as a safety ranking

The breadth of respiratory signals was greatest for ibrutinib in FAERS and smallest for pirtobrutinib, but these differences are confounded by market age and accumulated reporting. Pharmacologically, ibrutinib has broader kinase activity; acalabrutinib and zanubrutinib were developed with greater BTK selectivity; and pirtobrutinib is a highly selective non-covalent inhibitor (1, 2). These distinctions may influence immune, cardiovascular, and haemostatic effects, yet they do not provide a basis for translating spontaneous-reporting RORs into head-to-head clinical risk. The clinical pneumonia meta-analysis did not find significant incidence differences among the covalent agents (3). Likewise, the 2026 four-agent FAERS study described generation-specific reporting patterns but explicitly framed them as pharmacovigilance signals rather than comparative incidence (10). Our findings complement that work by focusing on respiratory phenotypes and testing replication in an independent Japanese database.

### 4.5 FAERS and JADER provided complementary evidence

Signal density differed markedly between the databases. FAERS contributed substantially more target-drug and respiratory-SOC records, whereas JADER was smaller, more physician-driven, and concentrated in older patients. Only 102 respiratory-SOC adverse-event records were available across the four JADER target-drug cohorts, compared with 12,911 in FAERS. The value of JADER is therefore not that every FAERS signal should reappear, but that concordant findings arise in a separate reporting environment. Replication of ibrutinib-associated epistaxis, haemothorax, and pleural effusion, and of pneumonia for three agents, strengthens confidence that these reporting patterns are not unique to one database. Conversely, non-replication in sparse strata—especially zanubrutinib and pirtobrutinib—remains inconclusive. This logic is consistent with recommendations to use conservative thresholds and count-aware interpretation in small pharmacovigilance subsets (17) and with recent dual-database work using FAERS and JADER (18). Non-replication of respiratory-SOC PT signals for the newer agents should be considered in the context of limited reporting opportunity in JADER. Zanubrutinib contributed only 54 JADER reports, all from 2025, and pirtobrutinib contributed 83 reports from 2024–2025. The former was first approved in the United States in November 2019 and in Japan in December 2024; the corresponding dates for pirtobrutinib were January 2023 and June 2024 (15, 19, 20). Thus, the interval between initial US and Japanese approvals was approximately five years for zanubrutinib and 17 months for pirtobrutinib. Sparse strata have lower sensitivity for meeting the prespecified minimum count and confidence-interval criteria, although no formal power calculation was performed. The absence of replication for individual respiratory-SOC PTs is therefore inconclusive and does not imply an absence of respiratory toxicity in Japanese patients. This qualification does not apply to the focused Pneumonia result for pirtobrutinib, which met the primary ROR criterion in both databases.

### 4.6 Time-to-onset patterns

TTO analysis should be interpreted separately from disproportionality. In FAERS, ibrutinib-associated pneumonia had a median TTO of 114.5 days and an early-failure Weibull pattern (beta=0.55, 95% CI 0.53–0.58). Acalabrutinib also showed an early-failure pattern in FAERS (median 112.0 days; beta=0.69, 95% CI 0.59–0.83). In JADER, the beta confidence intervals for ibrutinib and acalabrutinib included 1, corresponding to random-failure classifications; the acalabrutinib estimate was based on seven reports and is exploratory. The random-failure classifications in JADER should not be interpreted as evidence against an early treatment-related component. Only 36 ibrutinib and seven acalabrutinib pneumonia reports had valid TTO data in JADER, leaving substantial uncertainty, particularly for acalabrutinib. In these two JADER cohorts, patients were older and reports were predominantly submitted by physicians. Older age could contribute to sustained background susceptibility to infection, and differences in clinical case mix or reporting thresholds could attenuate an early concentration of reports. These remain hypotheses because comorbidities and other individual infection-risk factors could not be characterized comprehensively. Zanubrutinib showed an early-failure estimate in 13 FAERS reports, but this small sample likewise warrants caution. These results differ in emphasis from the interval-based temporal description in the 2026 FAERS study by Tu et al., which noted a larger proportion of some ibrutinib pneumonia reports at later treatment intervals (10). The analyses are not directly contradictory: our Weibull model uses the complete distribution of exact start-to-event intervals among reports with valid dates, whereas interval-based summaries describe the relative distribution of reported cases across predefined time bins.

Clinically, the data do not support a single fixed surveillance window. Early clustering in some FAERS strata suggests that vigilance is important soon after treatment initiation, but pneumonia also occurred at later times. TTO in spontaneous-reporting systems is conditioned on both event reporting and date completeness and cannot be interpreted as a population hazard over time.

### 4.7 Strengths and limitations

The study has several strengths. It included four clinically relevant BTK inhibitors, including the non-covalent agent pirtobrutinib; used two independent spontaneous-reporting systems; separated formal respiratory-SOC analysis from the prespecified Pneumonia PT; applied four complementary disproportionality algorithms for the focused pneumonia analysis; and used explicit cross-database replication and harmonized TTO definitions. Raw report counts, target-drug role restrictions, respiratory PT classifications, signal status, and temporal data were re-audited against the source exports before final analysis.

The limitations are those inherent to spontaneous reporting and are substantial. FAERS and JADER are affected by underreporting, stimulated reporting, missingness, duplicate or follow-up reporting, and differential reporting by drug age and clinical use. Exposure denominators are unavailable, so incidence, relative risk, and causality cannot be estimated. Confounding by indication is especially relevant because B-cell malignancies themselves predispose to infection, cytopenia, bleeding, and pulmonary complications. Previous therapies, corticosteroids, antimicrobial prophylaxis, concomitant anticoagulants or antiplatelet agents, and disease severity could not be comprehensively adjusted. Pneumonia is etiologically heterogeneous, and respiratory PTs such as pleural effusion or pulmonary oedema have multiple competing causes. Finally, JADER counts for newer agents and valid TTO counts for several drug–database combinations were small; extreme estimates from one or two reports were therefore not considered confirmed signals (17).

The exact MedDRA PT Pneumonia does not identify a causative organism or provide the clinical information needed for etiologic adjudication. We could not reliably distinguish bacterial, viral, fungal, or aspiration-related pneumonia, nor exclude diagnostic overlap or miscoding involving non-infectious inflammatory pulmonary injury. This limits translation of the signal into pathogen-specific management. Future studies linking pharmacovigilance reports with electronic health records, microbiology, imaging, and treatment data are needed for refined pulmonary phenotyping.

### 4.8 Clinical implications

The findings support practical respiratory surveillance rather than drug-specific risk ranking. Current infection-management guidance supports assessment of individual infection risk during BTK inhibitor treatment (21). Our temporal findings support heightened attention to respiratory infections in the early months after initiation, including the first three months, particularly in older or immunocompromised patients. Surveillance should continue thereafter because pneumonia was also reported at later time points; the spontaneous-report TTO distribution does not define a clinical risk cutoff.Pneumonia warrants attention throughout BTK inhibitor treatment, particularly in patients with relapsed/refractory disease, prior intensive treatment, corticosteroid exposure, cytopenias, hypogammaglobulinemia, or other markers of immune compromise (3–6). Respiratory symptoms should not automatically be attributed to infection: bleeding, inflammatory pneumonitis, cardiac-related pulmonary oedema, pleural malignancy, and other causes remain important alternatives. In patients with haemorrhagic manifestations, platelet count, concomitant antiplatelet or anticoagulant therapy, and procedural history should be reviewed (7–9). Prospective studies with exposure denominators and detailed clinical phenotyping are needed to determine whether the replicated reporting patterns correspond to measurable differences in clinical risk.

## 5 Conclusion

Four BTK inhibitors showed heterogeneous respiratory adverse-event reporting profiles in FAERS and JADER. Pneumonia was the most reproducible clinically important event, with cross-database ROR replication for ibrutinib, acalabrutinib, and pirtobrutinib. Within the formal respiratory SOC, replication was limited to ibrutinib-associated epistaxis, haemothorax, and pleural effusion. FAERS temporal data showed early-failure patterns for pneumonia with ibrutinib and acalabrutinib. By contrast, the JADER estimates exhibited less consistent temporal patterns and were often constrained by limited case counts. For newer BTK inhibitors, the failure to replicate individual respiratory PT signals within the SOC in JADER should be interpreted cautiously, as sparse reporting and shorter post-marketing exposure in Japan substantially limit statistical power and signal detectability. Accordingly, the absence of a replicated signal should not be construed as evidence of absent respiratory toxicity.These findings support continued respiratory and infection surveillance but should be interpreted as hypothesis-generating reporting patterns rather than evidence of incidence, comparative safety ranking, or causality.

## Data Availability

The original contributions presented in the study are included in the article/supplementary material. Further inquiries can be directed to the corresponding author.
